# Dementia prevention and the GP’s role: a qualitative interview study

**DOI:** 10.3399/BJGP.2023.0103

**Published:** 2023-08-08

**Authors:** Danielle Jones, Rachael Drewery, Karen Windle, Sara Humphrey, Andreia Fonseca de Paiva

**Affiliations:** Centre for Applied Dementia Studies, Faculty of Health Studies, University of Bradford, Bradford.; Centre for Applied Dementia Studies, Faculty of Health Studies, University of Bradford, Bradford.; Centre for Applied Dementia Studies, Faculty of Health Studies, University of Bradford, Bradford.; GP with an extended role in older people, associate clinical director frailty/dementia and LD, Bradford District and Craven Health and Care Partnership, Bradford; medical director, Westcliffe Health Innovations, Bradford; clinical lead, Yorkshire and the Humber Clinical Network (Dementia and Older Peoples Mental Health); honorary visiting professor, Faculty of Health Studies, University of Bradford, Bradford.; Centre for Applied Dementia Studies, Faculty of Health Studies, University of Bradford, Bradford.

**Keywords:** brain health, dementia, general practice, prevention, primary health care, risk reduction

## Abstract

**Background:**

GPs play an increasingly important role in proactively preventing dementia. Dementia in 40% of patients could be prevented or delayed by targeting 12 modifiable risk factors throughout life. However, little is known about how GPs perceive their role in dementia prevention and the associated barriers.

**Aim:**

To explore the role of GPs in dementia prevention.

**Design and setting:**

Qualitative study among UK GPs.

**Method:**

Semi-structured online interviews were conducted with 11 UK GPs exploring their views regarding their role in dementia prevention. Data were analysed using thematic analysis.

**Results:**

GPs reported that they never explicitly discuss dementia risk with patients, even when patients are presenting with risk factors, but acknowledge that dementia prevention should be part of their role. They advocate for adopting a whole team approach to primary care preventive practice, using long-term condition/medication reviews or NHS health checks as a platform to enable dementia risk communication targeting already at-risk individuals. Barriers included a lack of time and an absence of knowledge and education about the modifiable dementia risk factors, as well as a reluctance to use ‘dementia’ as a term within the appointment for fear of causing health anxiety. ‘Brain health’ was perceived as offering a more encouraging discursive tool for primary care practitioners, supporting communication and behaviour change.

**Conclusion:**

There needs to be a whole-systems shift towards prioritising brain health and supporting primary care professionals in their preventive role. Education is key to underpinning this role in dementia prevention.

## INTRODUCTION

Globally, dementia is one of the fastest- growing causes of death and disability, and it poses a significant social, health, and economic crisis.^1^ Dementia prevalence in the UK is rising, with one in 14 people aged >65 years living with dementia.^2^ Dementia research has predominantly focused on cure or care, much less so on the prevention of dementia.^3^

Twelve modifiable risk factors were presented by the 2020 *Lancet* Commission,^4^ encompassing: less education (in the earliest stages of life <45 years), hearing loss, traumatic brain injury, hypertension, excessive alcohol consumption, obesity, smoking, depression, social isolation, physical inactivity, air pollution, and diabetes. These account for around 40% of dementias in people worldwide. There is growing consensus that interventions targeted at reducing risks or modify risk behaviours may prevent or delay dementia.^4^

Recent guidance has highlighted the importance of dementia prevention. Area three of The World Health Organization’s global action plan (2017–2025)^1^ explicitly focuses on dementia risk reduction, detailing that health and social care professionals, especially those within primary care, should proactively manage modifiable dementia risk factors throughout the life course. This is echoed in UK policy through its national dementia strategies,^5^^–^8 public health policy,^9^ the NHS Five Year Forward View, and the NHS Long Term Plan.^10^^,^^11^

The NHS Long Term Plan^11^ proposes that within the new integrated care systems, primary care networks will adopt a proactive population health approach to enable earlier detection and intervention of at-risk populations, including those at risk of dementia. However, objectives for achieving this are often vague and generally bundled within advice relating to other non- communicable diseases,^12^ with research regarding their feasibility in practice inconclusive.

GPs could play a crucial role in dementia prevention and are increasingly identified as the preferred source of information.^13^^–^15 Primary care services already offer pathways for non-communicable disease prevention.^16^ As the risk factors for dementia are shared with other non-communicable diseases,^17^ with established programmes on risk reduction and health promotion (for example, weight management, smoking cessation, and physical activity^18^^–^20), primary care professionals are well placed to engage in preventive practice for dementia.

Dementia research in primary care has often focused on the GP’s role in diagnosing and managing dementia,^21^^,^^22^ rather than delivery of preventive advice. Primary care- based interventions have increased to target at-risk populations and promote healthier lifestyles.^23^^,^^24^ For example, one vehicle to support dementia prevention could be the UK’s NHS health check programme, delivering 5-yearly health checks to those aged 40–74 years.^25^ However, uptake has been lower than expected,^26^ and research on their utility is inconclusive.^27^^,^^28^

**Table:** How this fits in

There is growing consensus that interventions targeted to reduce risks or modify risk behaviours may prevent or delay dementia. Limited international research shows that although awareness of dementia risk factors is good, primary care professionals lack confidence in their knowledge, resulting in uncertainties in dementia risk reduction advice. This qualitative interview study is, to the authors’ knowledge, the first to explore the role of UK GPs in dementia prevention. Updating service delivery to include brain health in long-term condition/medication reviews or NHS health checks, and advising primary care professionals on how to communicate with patients about their brain health could help to promote healthy lifestyle changes to reduce the risk of dementia.

Although there is limited research on GPs’ views regarding their role in dementia risk reduction, an Australian survey^29^ found that 62% of healthcare providers reported that they had not received any training in dementia prevention. Furthermore, the study showed that although awareness of dementia risk factors was good, primary care professionals lacked confidence in their knowledge, leading to uncertainties in risk reduction advice.

Research in the US has identified the feasibility and acceptability of dementia screening programmes within primary care, noting that patients who recalled being counselled about modifiable risk factors were more likely to report positive health behaviour changes.^30^ Despite such findings, there remains little research in the UK exploring GPs’ views regarding their knowledge of modifiable dementia risk factors, the extent to which they discuss this with patients across the life course, and those barriers that may limit their engagement in dementia risk communication.^31^

This unique, to the authors’ knowledge, exploratory, qualitative study develops the evidence-base by investigating the GP’s role in dementia prevention. The objectives were to:

- examine UK GPs’ awareness of the modifiable dementia risk factors;
- identify if, when, and how dementia risk is communicated to patients; and
- explore the barriers and enablers to dementia risk communication in primary care practice.

## METHOD

Twenty-two GPs were invited to participate in a single recorded semi-structured, online interview. They were recruited through convenience sampling from existing networks in the UK. The sampling method was determined by the limited timescale of the project and ethical permissions granted. Invitations and the project information sheet were sent via email. Consent was gained in writing before the interview. Interviews were conducted by the first and last authors, both trained in qualitative methods and experienced dementia researchers. The interviews followed a topic guide developed by the research team (see Box 1) and reviewed and amended iteratively following the first interview as new topics were identified. Probes and follow-up questions elicited more detailed information, ensuring all topics were covered.

**Box 1.** Interview guide: overview of main topics and corresponding sample questions

**Topic**	**Sample question(s)/prompts**
1. Participant characteristics	How long have you been a GP? Have you received any dementia education and/or training? If so, what and when was this? Have you been involved in dementia research previously? If so, what and when was this?
2. Awareness of the modifiable dementia risk factors	Can you tell me what you know about the modifiable risk factors fordementia? When and how did you become aware of these modifiable risk factors?
3. Role in dementia prevention	Can you tell me about the role you play in preventing dementia? Under what circumstances would you discuss dementia risk with patients? Do you ever explicitly discuss dementia risk with your patients? Do you have any strategies for discussing dementia risk with patients? Do you think dementia prevention should be part of the GP’s role?
4. Barriers and enablers to dementia risk communication	Can you tell me about any barriers which may prevent you from discussing dementia risk with patients? Can you tell me what would help you to have these conversations about dementia risk with patients?
Added following Interview 1	
5. Brain health	Can you tell me what you understand about brain health? Do you think brain health could be a useful way to discuss dementia risk, if so, when would you use it and how?
6. Additional topics	Is there something else you would like to discuss?

Interviews were transcribed verbatim with identifiable information removed. Data were analysed using thematic analysis,^32^ managed by NVivo (version 12) software. The two interviewers independently familiarised themselves with the data and generated initial codes. These codes were then collaboratively organised into themes, which were reviewed and refined to accurately capture the data. The final set of themes were reviewed by all co-authors, named, and described in detail, with representative quotes selected to illustrate each theme. To enhance the validity of the analysis, the research team engaged in reflexivity throughout the analysis process, acknowledging assumptions and biases, and working to minimise their impact.^33^

## RESULTS

Eleven GPs were interviewed (*n* = 11/22 invited, 50% recruitment rate, recorded between July and November 2022). No new codes were identified in the final two interviews, with data saturation achieved, signifying an adequate sample size.^34^ The main reason specified for declining an interview was workload pressure. The average length of the interviews was 26 min. A total of 82% (*n* = 9/11) of participants were female, which is unrepresentative of GP workforce demographics.^35^

The cohort was dichotomised in terms of years of experience, being evenly split between ≤5 years and >20 years, delivering representation from both new and more experienced GPs. All participants had received some dementia education as part of their medical education or further continuing professional development, with over half of the participants receiving specialist postgraduate dementia education. Participant characteristics are presented in Table 1.

**Table 1.** Characteristics of participating GPs (*N* = 11)

**Characteristic**	**GP,** ***n***
**Sex**	
Male	2
Female	9

**Time qualified as GP, years**	
≤5	5
6–20	1
>20	5

**Highest level of dementia education**	
As part of medical education before GMC registration	1
Post-registration CPD dementia training	4
Post-registration postgraduate dementia education	6

**Prior dementia research involvement**	
Yes	1
No	10

*CPD = continuing professional development. GMC = General Medical Council.*

**Figure 1.**
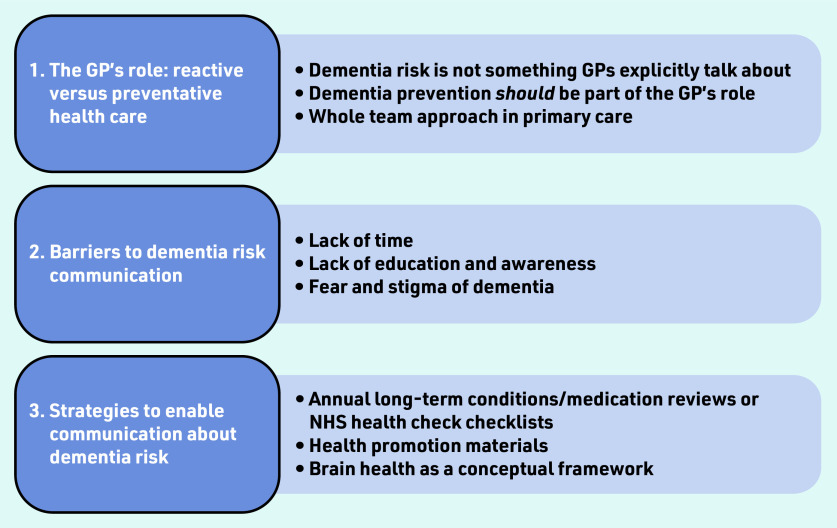
*Overview of themes.*

Three overarching themes were generated (Figure 1), identifying the current role GPs play in dementia prevention, their knowledge of dementia risk factors, and those barriers and future strategies suggested to ensure dementia risk communication appears in primary care discourse. The three themes were:

- the GP’s role: reactive versus preventive health care;
- barriers to dementia risk communication; and
- strategies to enable communication about dementia risk.

### Theme 1: the GP’s role: reactive versus preventive health care

Many of the participants discussed their role in the treatment of acute illness, as well as in the management and monitoring of chronic conditions, but less so their role in preventive medicine. None identified their role in screening for dementia. Overwhelmingly, discussion on preventive discourse focused on secondary prevention and in reducing further risk associated with the (mis)management of conditions such as diabetes, cardiovascular disease, and obesity. Although participants frequently advise about the future risks of stroke, heart attack, and cancer associated with these conditions, dementia does not appear as part of the risk reduction discourse:

*‘… we have to prioritise and often it’s the acute problems that have to come first.’*

(GP10)

*‘We think a lot about hearts and kidneys and diabetes, but maybe don’t think dementia prevention quite as much as we should.’*

(GP7)

#### Dementia risk is not something GPs explicitly talk about

All participants stated that they never explicitly discuss dementia risk with patients, even when patients present with risk factors:

*‘I never talk about dementia risk factors.’*

(GP1)

*‘We are regularly saying, oh your obesity, is putting you at risk of breast cancer, ovarian cancer … but we have not quite got into our pattern, saying you are at risk of having a heart attack, stroke and obesity, oh and by the way, you are at risk of dementia.’*

(GP6)

Only two participants identified an exception, when the patient raises dementia as a particular concern, or it appears in the patient’s family history and may be a contributory factor to current medical concerns:

*‘No, I don’t think I do and that unless it’s come up through some other reasons. So if they’ve talked about a family history of dementia or something, then I’ll be much more likely to* [talk about dementia risk]*.’*

(GP4)

#### Dementia prevention should be part of the GP’s role.

Most participants acknowledged the need to play an active role in dementia prevention, engaging in communication about dementia risk. One GP went further to state that having been involved in the research and becoming more aware of dementia risk factors, they had started introducing dementia risk into their clinical practice.

Importantly, some of the participants noted that risk communication related to other non-communicable diseases (for example, smoking and smoking cessation) is becoming ineffective. It was suggested that adding dementia into risk discourse may have a greater impact in influencing behaviour change:

*‘... sometimes you hear so much smoking is bad for your lungs. Like it doesn’t mean anything anymore. Whereas maybe if you just start saying actually smoking also can increase your risk of getting dementia, that might be something that sticks with them more. So I think it is worth saying, yeah, I think it can have an impact.’*

(GP12)

*‘... if someone came in and had high blood pressure … and we’d recommend starting medication and the reason being is that you know if you have high blood pressure you* [are] *at greater risk from strokes and heart attacks. And in the future if I was going to then say about dementia … actually this ties into what we call cardiovascular disease, which is the health of your blood vessels, which has greater impact on things like your kidneys, but also your brain. And I did start doing that with a patient last week after having the conversation with* [researcher name]*.’*

(GP9)

#### A whole team approach in primary care

Most of the participants identified that a greater impact could be achieved in dementia prevention if a ‘whole team approach’ was adopted. For example, although physician associates, nurse practitioners, pharmacists, advanced paramedics, and other allied health professionals played a key role in annual long-term condition/medication reviews and NHS health checks, participants were uncertain if dementia was discussed with patients during these appointments.

To ensure patients were supported to reduce their dementia risk, GPs advocated for the inclusion of the wider primary care team in any future research, education, or implementation of future interventions:

*‘... it’s the role of a GP or the GP practice as a wider team to manage those risks factors.’*

(GP8)

*‘I think just the involvement of the whole practice team … a lot of the prevention work or chronic disease monitoring is done by the nurses or allied health professionals. So we know in our practice we’ve got an advanced paramedic and he does … the majority of our frailty work. Everyone is key for primary care, and because it’s very much a shared role.’*

(GP9)

Along with a whole primary care team approach, some participants looked to the third sector and public health messaging to play a role in the dementia prevention agenda:

*‘And the Alzheimer Society, all those kinds of things they tell them about this. So it probably doesn’t mainly come from the GP. I would say it’s more the charities and the extra support services that are there and carers resources.’*

(GP8)

*‘Actually in the real world, I think it’s much more of a public health message that should be taken on, you know, we’d be supported but should be taken on by Public Health England.’*

(GP4)

### Theme 2: barriers to dementia risk communication

Participants identified three fundamental barriers to dementia risk communication with patients: a) lack of time; b) lack of education and awareness of the dementia risk factors and how to communicate these to patients; and c) the fear and stigma of dementia.

#### Lack of time

All participants stated that a lack of time prohibited discourse on dementia prevention. Often the mention of dementia led to a protracted conversation, further having an impact on service delays:

*‘GPs unfortunately don’t have the time.’*

(GP12)

*‘As you’re probably well aware of time pressures, having 10 minutes, it’s really challenging to fit everything in ... sometimes a certain phrase can almost open a can of worms … And it’s almost easier, sometimes not to open the can of worms.’*

(GP10)

#### Lack of education and awareness

A further barrier is a lack of education and knowledge regarding the modifiable dementia risk factors. When asked (see Box 1), participants demonstrated good awareness of the cardiovascular risks associated with vascular dementia and identified many of the modifiable dementia risk factors (as per the 2020 *Lancet* Commission^4^) (see Table 2). However, there were absences in their knowledge, including the risks associated with having less education on health risks, hearing loss, traumatic brain injury, and air pollution. Many participants admitted to feeling unaware of or ill- prepared to discuss dementia risks:

*‘I think there is work to be done, and I think really that’s about just updating people’s knowledge, encouraging people to go on training and updates.’*

(GP6)

**Table 2.** GP awareness of modifiable risk factors for dementia

**Modifiable risk factor**	**Identified by GP, *n***
Less education	2
Hearing loss	2^a^
Traumatic brain injury	0
Hypertension	11
Alcohol >21 units/week	11
Obesity	11
Smoking	11
Depression	5
Social isolation	5
Physical inactivity	8
Air pollution	1^a^
Diabetes	11

a *One GP worked in the community with the ear, nose, and throat team and one GP read the 2020* Lancet *Commission^4^ as part of their postgraduate dementia education on the morning of the interview.*

#### Fear and stigma of dementia

The term ‘dementia’ inhibits discussions about dementia risk. Most participants identified that given dementia engenders anxiety in patients and is still a stigmatised condition, they avoided introducing it within the clinical consultation:

*‘They’re more worried about it* [dementia]*. I think it’s overtaking cancer as the number one worry and a lot of people who are kind of frightened of it.’*

(GP4)

*‘I think traditionally we haven’t necessarily talked about it because there’s still some stigma attached to the word dementia … So I think that there is a stigma. I think it is scary, but do I think that means we shouldn’t use it — absolutely not.’*

(GP5)

### Theme 3: strategies to enable communication about dementia risk

Participants identified strategies to enable their role in dementia prevention. These strategies involved policy and service delivery amendments and conceptual considerations about the terminology of dementia and the utility of ‘brain health’ — an evolving concept becoming increasingly popular (for a fuller definition see Kolappa *et al*)*.*^36^

#### Health reviews checklists

Most participants discussed placing dementia risk communication in the annual long- term condition/medication reviews for patients with other non-communicable diseases, and the NHS health checks. They emphasised that dementia would only be discussed at these reviews if added to the checklists used by the primary care team:

*‘A lot of the reviews are done by the nurses, and they have tick boxes, there’s quite a lot of bureaucracy in general practice and there’s a lot of tick boxes to get funding. So if it was incorporated within that then it becomes something more.’*

(GP9)

*‘I think from my point of view as a GP, in this practice, I should keep encouraging my nurses, who do most of this work, frankly, to mention it as part of that screening in the health check.’*

(GP6)

#### Health promotion materials

To facilitate their role in a time pressured environment, two participants suggested that health promotion materials could be necessary (for example, leaflets or signposting people to web pages):

*‘… even a leaflet to give to patients. You know, what’s dementia? How can I prevent it? Something like that might be good.’*

(GP12)

*‘I guess you don’t have to communicate everything during the consultation, do you? You can signpost people off to other relevant information where they can choose to watch it at their leisure.’*

(GP7)

#### Utility of the concept and terminology of brain health

While use of the term dementia was perceived to cause patients anxiety, and protract the length of appointments, the concept and terminology of brain health was perceived by most participants as a useful and more encouraging narrative, supporting communication, behaviour change, and medication adherence:

*‘I think it* [brain health] *would be a really useful promoting thing because it’s a positive thing, isn’t it? It’s a positive message.’*

(GP6)

*‘And I quite like it* [brain health] *… I think that could feed into people’s understanding and actually getting them to be more likely to take their medications.’*

(GP9)

*‘I think traditionally we haven’t necessarily talked about it because there’s still some stigma attached to the word dementia. So I think that’s why brain health is useful.’*

(GP5)

## DISCUSSION

### Summary

The GP’s role is dominated by treating acute illness and managing chronic conditions, but less so in preventive medicine. Preventive discourse in GPs’ clinical communication focuses on secondary prevention, reducing further risk associated with the (mis)management of long-term conditions. Although GPs frequently advise about the future risks of stroke, heart attack, and cancer, dementia does not appear as part of the risk or risk reduction discourse. Consequently, participants reported that they never explicitly discuss dementia risk, even when patients present with known modifiable risk factors. Despite this, participants unanimously agreed that they have a significant role to play in dementia prevention. Some went further to suggest that dementia risk communication could ‘freshen up’ the risk advice about other non-communicable diseases, which is at times ineffective in delivering medication adherence or lifestyle behaviour change.

Participants demonstrated good awareness of the cardiovascular risks associated with dementia, identifying many of the modifiable dementia risk factors. However, there were noticeable absences, including the risks associated with having less education, hearing loss, traumatic brain injury, and air pollution.

Many participants admitted to feeling ill-prepared to discuss dementia risks with patients. Further barriers that inhibit the role GPs play in dementia prevention include a lack of time and the ongoing stigma and health anxiety caused by the mention of dementia.

Brain health could be a more positive framework to use when discussing dementia risk, potentially motivating behaviour change or adherence to medication regime. While dementia continues to be stigmatised, brain health offers a more encouraging narrative to aid communication about dementia risk.

To put dementia prevention on the primary care agenda, brain health needs to be added to NHS health check templates, and annual long-term condition/medication review checklists, ensuring that conversations about dementia risk occur during these appointments. One participant identified that financial incentives for dementia prevention interventions, akin to those provided through the Quality and Outcomes Framework (QOF)^37^ for other conditions (such as diabetes and heart disease), may change clinical behaviour. However, there is limited evidence of applying the QOF as a ‘lever’ for change.^37^

Further research, education, and intervention about dementia prevention needs to adopt a whole primary care team approach, exploring the role other health professionals play within the primary care context.

### Strengths and limitations

This study provides unique insights into the views of GPs regarding their role in dementia prevention, and evidence of their awareness (or lack of awareness) of modifiable dementia risk factors. It has identified barriers that inhibit dementia risk communication in primary care practice, but also highlighted strategies to facilitate GPs in their role in dementia prevention.

Convenience sampling led to half of the participants having (or undertaking) postgraduate dementia education, working towards extended role status.^38^ As a result, the participants perhaps demonstrated more advanced knowledge of the topic area than most GPs, limiting transferability of the findings. However, when compared, there was remarkable similarity in the views of participants with and without specialist dementia training, in relation to current practice, barriers, and strategies proposed to enhance their role in dementia prevention.

A more comprehensive sampling strategy involving the wider primary care team, midcareer, and male GPs, as well as obtaining views from a range of community practices, could have enhanced this study. Considering correlations between demographic data, prior training and experience, and GPs’ perspectives may have further strengthened the analysis.

### Comparison with existing literature

Echoing prior research,^29^ there are system-level factors inhibiting the GP’s role in dementia prevention: the focus on treatment of acute illness, the management of chronic conditions, time restraints during clinical appointments, and a lack of confidence in how to introduce dementia risk in clinical discourse.

Although Zheng and colleagues^29^ noted that primary care professionals had good knowledge of the dementia risk factors, in part reflected in this study, there are noticeable absences. Education for GPs and wider primary care teams is advocated in both policy^6^ and research,^39^ and is indicated in this study as being an important intervention to enhance the role of primary care professionals in the prevention of dementia.

Recent guidance promotes the role of primary care in adopting a proactive population health approach to deliver earlier intervention to at-risk populations. Alzheimer’s Disease International^40^ recommends introducing annual brain health check-ups for those aged ≥50 years, providing focused opportunities to promote dementia risk reduction.^40^ One study has noted the feasibility of such screening interventions.^30^ However, in UK-based population studies, the most common recommendation was to embed proactive approaches in routine health check-ups, for example, the annual health review of older adults.^27^ GPs in this study mirrored this suggestion and would require further investigation.

The power of language in medical discourse is widely discussed.^41^^,^^42^ With dementia being stigmatised,^43^ and considering some communities do not have a word for dementia,^44^ attention should be paid to the language used for healthcare advice. Brain health, as previously mentioned, is an evolving concept that has become increasingly popular*.*^36^ Findings from the interviews mirror research that suggests that brain health could be a useful discursive tool to enable communication about risk factors associated with dementia.

Alzheimer’s Research UK^13^ showed that 69% of UK adults believe they can influence their brain health, whereas only 34% believe they can reduce their risk of developing dementia. Although there is evidence to suggest that health promotion should not focus specifically on dementia but instead prioritise a healthy lifestyle more broadly,^27^ adopting a healthy ageing approach using brain health could be a promising narrative to transform how people think and communicate about dementia risk over the life course and across communities. Brain health could also reinvigorate conversations about risks associated with other non-communicable diseases where the risk discourse may have become repetitive and ineffective at motivating behaviour change.^14^

There needs to be consideration about how this discourse is enacted to avoid adverse effects on patient resistance to advice,^45^ as well as mitigating blame discourse around the self-responsibility for dementia.^41^ The utility of brain health in primary care dementia risk discourse warrants further examination.

### Implications for research and practice

Implementing a brain health discourse approach to facilitate risk reduction, related to both dementia and other non- communicable diseases, could result in positive communications and health outcomes.

Adopting a whole team approach within primary care, using annual long-term conditions/medication reviews as a site for brain health communication, could overcome some of the barriers identified. Primary care teams should ensure that dementia appears on the checklists used in these appointments.

An evaluation of this intervention, applied throughout the life course and across communities, measuring its impact on behaviour change is necessary.

With primary care in England expanding^11^ to include practitioners with advanced clinical skills as well as 17 new roles under the additional roles reimbursement scheme,^46^ additional research needs to be undertaken to understand the prevention agenda within these new multidisciplinary teams.

There needs to be a whole-systems shift towards prioritising brain health and supporting primary care professionals in their preventive role. Education is key to underpinning this role in dementia prevention, with a sharper focus on brain health needed in pre-registration education programmes.

Educational interventions must be strongly embedded in policy guidance and appear on subject benchmarks and curricula. Primary care practice requires support through clear public health policy and messaging on brain health to ensure awareness of dementia risk is received at a population level.
